# Recombinant rabbit beta nerve growth factor production and its biological effects on sperm and ovulation in rabbits

**DOI:** 10.1371/journal.pone.0219780

**Published:** 2019-07-18

**Authors:** Ana Sanchez-Rodriguez, Paloma Abad, María Arias-Alvarez, Pilar G. Rebollar, José M. Bautista, Pedro L. Lorenzo, Rosa M. García-García

**Affiliations:** 1 Department of Physiology, Faculty of Veterinary Sciences, Complutense University of Madrid, Madrid, Spain; 2 Department of Biochemistry and Molecular Biology, Faculty of Veterinary Sciences, Complutense University of Madrid, Madrid, Spain; 3 Department of Animal Production, Faculty of Veterinary Sciences, Complutense University of Madrid, Madrid, Spain; 4 Department of Agrarian Production, ETSIAAB, Polytechnic University of Madrid, Madrid, Spain; University of Hyderabad, INDIA

## Abstract

In some induced-ovulating species, beta nerve growth factor (β-NGF) has important roles in ovulation, though data for rabbits are still inconclusive. In this study we first synthesized functional recombinant β-NGF from rabbit tissue (rrβ-NGF) to address the following objectives: 1) to compare rabbit β-NGF amino acid sequence with those of other induced- or spontaneous-ovulating species; 2) to assess the effects of rrβ-NGF on rabbit sperm viability and motility, and 3) to examine the *in vivo* ovulation inducing effect of rrβ-NGF added to the seminal dose in rabbit does. The *NGF* gene in rabbit prostate tissue was sequenced by Rapid Amplification of cDNA Ends and annotated in GenBank (KX528686). Recombinant rβ-NGF was produced in CHO cells and purified by affinity chromatography. Once confirmed by Western blotting and mass spectrometry (MALDI-TOF) that the amino acid sequence of the recombinant protein corresponded to β-NGF, its functionality was validated in PC12 cells in a successful dose-response study over 8 days. The amino acid sequence of prostate rabbit NGF differed to that of other species mainly in its receptor binding sites. In all the spontaneous ovulating species examined, compared with rabbit, alanine and proline residues, which interact with the high-affinity receptor, were replaced by a serine. In rabbits, asparagine and methionine were substituted by lysine at the low-affinity receptor binding site. In time- and dose-response experiments, the *in vitro* addition of rrβ-NGF to the ejaculate did not affect sperm viability whereas sperm motility parameters were enhanced by the addition of 1 μg/mL of the neuropeptide. Addition of this same concentration of rrβ-NGF to the seminal dose administered via the intravaginal route in does induced ovulation with a delayed LH peak, leading to a plasma progesterone increase, gestation and delivery. Our findings suggest that rrβ-NGF could be a useful option for biotechnological and reproduction assisted techniques in rabbits but further studies are needed.

## Introduction

Beta nerve growth factor (β-NGF) was first described as a neurotrophin with a regulatory role in the survival and maintenance of sympathetic and sensory neurons [1]. However, in more recent studies this neuropeptide has been also identified as an ovulation-inducing factor (OIF) present in the seminal plasma (SP) of some reflex ovulating species such as camelids or rabbits [2–5]. In rabbits, the neuropeptide and its receptors (TrkA and p75) have been identified in all male and female reproductive tissues (testes, epididymis and accessory glands [6–10], SP [7, 8, 10, 11], uterus, oviduct and ovary [12–14]). While some studies have suggested a role of β-NGF in the mechanism of ovulation in rabbits [8, 12, 15], evidence of its role as an OIF in this species, as found in camelids, is still scarce. Further, while the intramuscular administration of mouse β-NGF [8] or rabbit SP [3, 16] has been unsuccessful for inducing ovulation, an ovarian response in terms of increased numbers of hemorrhagic follicles (non-ruptured blood-filled anovulatory follicles) has been observed. In contrast, rabbit SP has been able to elicit ovulation in llamas [3], which suggests different ovulation patterns among reflex ovulators. Furthermore, ovulation in rabbits may be induced by nervous stimuli (mating) and an OIF present in semen [17]. While β-NGF is a highly conserved protein among species [18], the use of a specific recombinant rabbit β-NGF in homologous species could help our understanding of its role as an OIF.

Beta-NGF is synthesized as a precursor (pro-NGF), which is a 241-residue protein consisting of a signal peptide of 18 residues, a pro-peptide of 103 residues, and the mature form of 120 residues at the C-terminal end (data extrapolated and generalized from human β-NGF: http://www.uniprot.org/uniprot/P01138). N-glycosylation of pro-NGF is essential for processing, secretion and construction of the tertiary fold of the β-NGF homodimer [19, 20]. The correct folding of this homodimer is crucial for the protein's biological activity [21]. Moreover, post-translational modifications to the molecule, i.e. three disulfide bonds and a cysteine knot within the two β-NGF chains, are essential for its correct structure [22]. As all these modifications can only be efficiently generated via intracellular engineering of mammalian cells, Chinese hamster ovary (CHO) cells have been used for the recombinant production of biologically active human and mouse β-NGF [20, 23]. To evaluate the biological activity of recombinant β-NGF, researchers have extensively used a cell line derived from pheochromocytoma of the rat adrenal medulla (PC12 cells) as the β-NGF high-affinity receptor, TrkA, is present on the cell surface [24]. In effect, these cells respond to β-NGF by changing from proliferating chromaffin-like cells to non-dividing sympathetic neuron-like cells that develop axons or neurites [25].

In some species, β-NGF has been detected in semen, but its role in sperm physiology has been barely addressed. The neurotrophin has been localized in round spermatids and spermatocytes in the reproductive tract of the mouse and rat [26, 27] and it is thought to play a role in sperm differentiation [28, 29]. In ejaculated sperm, β-NGF promotes sperm motility and viability in humans [30–32], bulls [33] and golden hamsters [34], and also facilitates the acrosome reaction on caudal epididymal sperm [34]. In rabbits, no study has examined the effects of β-NGF on the main characteristics of ejaculated semen.

Nowadays, the administration via i.m. of GnRH analogues in the moment of the artificial insemination (AI) is necessary in the rabbit farms to elicit the ovulation. In order to avoid excessive handling and to improve animal welfare, new insights in ovulation induction by intravaginal route are being studied, such as the addition of synthetic GnRH analogues in the seminal doses. In fact, some studies have shown the effectiveness of these analogues (buserelin, triptorelin, lecirelin) to provoke the ovulation via intravaginal but higher dose than that required using other administration routes (approximately 10 times greater than the i.m. via) [17, 35–37]. In addition, it is reported that these higher doses reduce kindling rate in comparison with the i.m. GnRH administration [38]. Therefore, the intravaginal application of some molecules present in rabbit SP, such as β-NGF, could be an interesting approach to replace the high doses of these synthetic hormones when used intravaginally.

Specific substitutions in the amino acid sequence of rabbit β-NGF could condition the physiological actions of β-NGF. Thus, a homologous recombinant rabbit β-NGF could help clarify the specific role of this multi-functional protein as an OIF in this species. In this study, we examined whether adding exogenous β-NGF to the semen dose for AI would be useful by assessing effects on semen and ovulation. Our objectives were: 1) to produce and purify recombinant β-NGF from rabbit prostate tissue and confirm its biological activity in PC12 cells, 2) to compare β-NGF amino acid sequences in the rabbit and several representative species of induced- and spontaneous ovulation species, 3) to assess the effects of recombinant β-NGF on sperm viability and motility in ejaculated rabbit semen and, finally, 4) to test if adding this recombinant protein to the insemination dose will induce ovulation, conception and delivery in rabbit females. In this report, we describe the successful production and purification of recombinant rabbit β-NGF, its effects on sperm viability and motility, its capacity to induce ovulation and its main amino acid differences with the NGF from other species.

## Experimental procedures

### Production of recombinant rabbit β-NGF and its functional assessment in PC12 cells

#### Animals, tissue extraction and processing

New Zealand White x California adult male rabbits were housed individually in flat-deck cages with a light program consisting of 16 h of light and 8 h of darkness. Temperature was kept at 20 to 25°C and relative humidity at 60 to 75% by a forced ventilation system. Each animal had free access to food and water. All the experimental procedures with animals were approved by the Animal Ethics Committee of the Polytechnic University of Madrid (UPM, Spain), and were in compliance with the Spanish guidelines for care and use of animals in research [39].

Animals (n = 3) were euthanized and subjected to a ventral midline laparotomy. The prostate complex as described by Holtz and Foote [40] was dissected, the proprostate (localized at the cranial part of the prostate complex) was discarded, and the prostate recovered. Portions of 5 mm^3^ were collected in 1.5 mL tubes containing RNA later Stabilization Solution (Ambion, Thermo Fisher Scientific, Washington, USA) to avoid RNA degradation. RNA later was removed from tubes after maintenance at 4°C overnight and samples were stored at -80°C.

#### Complementary DNA sequencing of rabbit prostate β-NGF

To the best of our knowledge, the cDNA sequence of rabbit prostate *NGF* has not been reported previously. Total RNA was isolated using TRIzol reagent (Life Technologies, Thermo Fisher Scientific, Washington, USA), and mRNA was then obtained with FastTrack MAG mRNA Isolation Kit (Ambion, Thermo Fisher Scientific, Washington, USA) according to the protocol provided by the manufacturer. Next, cDNA was synthesized using a mix of random hexamers (0.5 μg/μL) and oligo (dT) primers (0.1 μg/μL) (SuperScrip First-Strand Synthesis System for RT-PCR, Life Technologies, Thermo Fisher Scientific, Washington, USA).

To sequence the entire *NGF* gene, specific primers were designed (Table 1) for a highly conserved region of the gene among species. For primer design, we took into account known alternative splicing of *NGF*, aligning amino acid and nucleotide sequences of different species (Clustal Omega Software and Serial Cloner 2.6 Software) to identify the conserved region.

**Table 1:** Specific primers for the conserved β-NGF region.

Primer β-NGF	5’– 3’ sequence	Tm (°C)	Multiplex (Kcal/mol)	Product length (nucleotides)
Forward	AGCCCACTGGACTAAACTGCA	61.3	-1.88	305
Reverse	TCGCACACCGAGAACTCTCC	62.5		

Tm: melting temperature (theoretical) calculated by OligoAnalyzer software.

Multiplex: complementary enthalpy between both primers.

Polymerase chain reaction (PCR) was performed using 1 μL of cDNA as a template for *β-NGF* specific primers, using the Platinum® Taq DNA Polymerase kit (Invitrogen, Thermo Fisher Scientific, Washington, USA). Cycling conditions consisted of an initial stage of 3 min of denaturation at 95°C, followed by 40 cycles of 30 seconds at 95°C, 30 s at 55°C and 15 s at 72°C, and a final elongation stage of 5 min at 72°C. Negative controls without reverse transcriptase and without DNA were run to rule out genomic DNA contamination. A 2% agarose gel was used to visualize the size of bands of the PCR products (10 μL per lane) using a scanner (Bio-Rad Laboratories, California, USA). The amplified products of 305 pb were purified from the agarose gel with SpeedTools PCR Clean-up kit (Biotools, B&M Labs, S.A., Madrid, Spain) and sequenced by the Sanger method using the BigDyeTM Terminator v3.1 Cycle Sequencing Kit (Thermo Fisher Scientific, Washington, USA) in a 3730XL DNA Analyzer sequencer (Applied Biosystems, Thermo Fisher Scientific, Washington, USA).

Once the conserved region of *NGF* was sequenced, the Rapid Amplification of cDNA Ends (RACE) procedure was used to obtain 5’ and 3’ sequences based on Frohman et al. [41] using SMARTer RACE 5’/3’ kit (Clontech Laboratories, California, USA). Inner and outer primers were designed (Table 2) and PCR cycling conditions (melting temperature of 68°C) were as indicated in the kit protocol.

**Table 2:** Specific primers designed for the rapid amplification of cDNA ends (RACE) method.

Primer	5’– 3’ sequence	Tm (°C)
3’ outer	GGGCAGACCCGCAACATCACCGT	70
3’ inner	CCCCAGACTTTTTAAGAAACGACGCCTG	70.1
5’ outer	TCGCACACCGAGAACTCTCCCATGTG	71
3’ inner	GTCCACCTCCAGGTCCAGCTCCT	70

Tm: melting temperature (theoretical) calculated by OligoAnalyzer software.

#### Plasmid production and transfection

The resultant rabbit prostate *NGF* sequence (KX528686) with a 7 x histidine tag at the C-terminal end was inserted in a pD2539-CEF plasmid with kanamycin and puromycin resistance genes (DNA 2.0, California, USA). The plasmid was produced in large quantities in *E*. *coli* HST08 cells (Stellar Competent Cells, ClonTech Laboratories, California, USA) incubated in LB medium containing 25 μg/mL of kanamycin at 37°C for 24 h with shaking. Plasmids were isolated by Megaprep (PureLink HiPure Plasmid Megaprep Kit, Thermo Fisher Scientific, Washington, USA) and then precipitated by the isopropanol/ethanol method. The resulting DNA was cut with the restriction enzyme Sal I to linearize the plasmid and then transfected with Lipofectamine 2000 Reagent (Invitrogen, Thermo Fisher Scientific, Washington, USA) into Chinese hamster ovary (CHO) cells (ATCC, Virginia, USA).

#### CHO cell culture and protein purification

Transfected CHO cells were first cultivated in F12 medium supplemented with HEPES 25 mM (Gibco, Thermo Fisher Scientific, Washington, USA), 10% fetal bovine serum (FBS, Gibco One Shot FBS, Thermo Fisher Scientific, Washington, USA), 125 μg/mL gentamicin (Invitrogen, Thermo Fisher Scientific, Washington, USA) and 5 μg/mL puromycin (Thermo Fisher Scientific, Washington, USA). Next, 500,000 viable cells/mL were subcultured in a T-160 cc flask (Thermo Fisher Scientific, Washington, USA) in serum free medium (CHO-S-SFM II, with hypoxanthine and thymidine, Thermo Fisher Scientific, Washington, USA) supplemented with 50 μg/mL gentamicin and 5 μg/mL puromycin for 4 days on a shaker platform. All cell cultures were placed in a humidified 5% CO_2_ atmosphere in an incubator (NuAire, Minnesota, USA) at 37°C.

Recombinant rabbit β-NGF (rrβ-NGF) was purified from the culture medium by affinity chromatography using Nickel columns (HisXL-Column High Density NICKEL, Agarose Bead Technology, Florida, USA) by selecting only those proteins with the histidine tag. Columns were equilibrated with 5 bed volumes of binding buffer (20 mM disodium phosphate, 500 mMNaCl, 10 mM imidazole at pH 7.5) and the culture medium was added so that it made contact with the resin for 15 min. After several washes of the column with binding buffer, the protein was then eluted in 10 mL of elution buffer (20 mM Disodium phosphate, 500 mM NaCl, 500 mM imidazole) and dialyzed in HEPES 10 μM for the PC12 bioassay, or in phosphate buffer saline 0.01M (PBS tablet, Sigma-Aldrich, Missouri, USA) to maintain semen viability for the rabbit sperm bioassay. Dialysis was performed by shaking at 4°C, replacing the medium 3 times after a minimum of 3 h of dialysis each time. Protein concentrations were measured by the Bradford method [42].

#### Western blot

To confirm the presence of rrβ-NGF, the dialyzed protein was subjected to TCA-acetone precipitation and then denatured in loading buffer (0.312 M Tris-HCl, 10% SDS, 25% 2-mercaptoethanol, 0.01% bromophenol blue, 50% glycerol) at 95°C. The denatured protein was then loaded on 12% SDS–PAGE gels which were run at 90 V for 2 h in duplicate. One gel was stained with Coomassie brilliant blue G-250 (Sigma Aldrich, Missouri, USA) and the other was used to transfer the protein to a nitrocellulose membrane (Ammersham Hybond ECL Nitrocellulose Membrane, GE Healthcare Life Science, Barcelona, Spain) (80 mA per membrane for 80 min). The membrane was blocked for 1 h with Odyssey blocking buffer (LI-COR Biosciences, Nebraska, USA), and then incubated at 4°C overnight with 0.1 μg/mL of goat anti-NGF antibody (N8773, Sigma-Aldrich) in blocking buffer with 0.1% Tween 20. After 6 washes, membranes were incubated at room temperature (RT) for 1 h with secondary antibody (IRDye 800CW Donkey anti-goat IgG (H + L), LI-COR Biosciences, Nebraska, USA). Finally, after additional washes, the membranes were scanned with an Odyssey fluorescence scanner (LI-COR Bioscience, Nebraska, USA).

#### MALDI-TOF mass spectrometry

For mass spectrometry analysis, the synthesized protein was subjected to SDS-PAGE and Coomassie staining as described above and gel bands of 13–15 kDa manually excised. The experimental procedure has been described elsewhere [8]. For protein identification, the sequence of rabbit prostate NGF (KX528686) was searched using MASCOT v 2.3 (www.matrixscience.com) via the Global Protein Server v 3.6 from ABSCIEX.

#### PC12 cell culture

For PC12 cell culture, cells were thawed at 37°C, centrifuged at 1800 x *g* 5 min to remove the cryoprotectant DMSO and then plated in a T-75 cc flask. Next, the cells were cultured in Dulbecco Modified Eagle Medium (DMEM, high glucose, HEPES) supplemented with 0.2 mM pyruvate, 10% horse serum (heat inactivated, New Zealand origin), 5% FBS and 50 μg/mL of gentamicin. All reagents were purchased from Thermo Fisher Scientific (Washington, USA). The medium was replaced every 48 h, and cells were incubated in a humidified atmosphere containing 5% CO_2_ at 37°C. PC12 cells were seeded at a density of 15,000 cells/500 μL in 24-well plates and grown for 24 h in an incubator at 37°C. The culture medium was supplemented with different concentrations of rrβ-NGF diluted in HEPES 10 μM: 0, 5, 10, 25, 50 and 100 ng/mL (0, 0.37, 0.74, 1.9, 3.7 and 7.4 nM) respectively and replaced every 48 h. Each rrβ-NGF dose was tested in triplicate and each experiment was repeated 3 times.

#### MTT assay

To examine the possible cytotoxicity of rrβ-NGF treatment in PC12 cells, we assessed cell viability at 48 h in a 3-(4,5-dimethylthiazol-2-yl)-2,5-diphenyltetrazolium bromide (MTT, M6494, Thermo Fisher Scientific, Washington, USA) assay. After discarding the media from wells, 200 μL of 500 μg/mL MTT in Locke medium (140 mM NaCl, 4.5 mM KCl, 2.5 mM CaCl_2_, 1.2 mM KH_2_PO_4_, 1.2 mM MgSO_4_, 5.5 mM glucose, 10 mM HEPES) were added to each well and these incubated for 2 h at 37°C. Next, 200 μL of solubilization buffer (0.1 M HCl, 1% Triton X-100 in isopropanol) were added followed by incubation for 1 h at RT to solubilize the formazan crystals. Sterile cell scrapers (Lab Clinics, Barcelona, Spain) were used to scratch the wells and the volume of each well was collected in tubes containing 0.9 mL of distilled water. The optical density (OD) of each sample was then measured at 560 nm using an UltroSpec III spectrophotometer (Pharmacia LKB, GE Healthcare Life Science, Barcelona, Spain). Data were analyzed in terms of the percentage of cell viability, calculated using the equation: (OD treated cells / OD non treated cells) x 100.

#### Neurite differentiation and outgrowth

At the moment of the bioassay, PC12 cells were plated in wells pre-coated with 7 μg/cm^2^ of collagen type IV (C6745, Sigma-Aldrich, Missouri, USA) at the same concentrations of rrβ-NGF eluted in HEPES 10 μM as used before. In each concentration plate, 5 images of a minimum of 100 cells were taken with a light microscope (Leica F550, Wetzlar, Germany) equipped with phase contrast optics and a DCF400 camera (Leica). The morphological differentiation of these cells was assayed by determining the percentage of differentiated cells (loss of round shape), the percentage of cells with neurite elongations and by measuring the length of the longest neurite per cell on Day 8 using ImageJ software (https://imagej.nih.gov/ij/) according to Haas et al. [43]. Cell elongations were considered neurites when their length was at least one cell diameter [43].

#### Immunofluorescence against anti-β-III tubulin

The neuronal differentiation of PC12 cells supplemented with rrβ-NGF was evaluated by indirect immunofluorescence against β-III tubulin, a microtubule element of the tubulin family found almost exclusively in neurons [44]. First, PC12 cells were cultured in 24-well plates in DMEM, supplemented as described above, on cover slips treated with polylysine (Biochrom, Cambridge, UK). The optimal concentration of rrβ-NGF identified earlier (25 ng/mL) was added 24 h after the beginning of cell culture and refreshed every 48 h. On Day 8 of treatment, the medium was removed and cells were washed with 0.1M PBS (16 mM NaH_2_PO_4_·2H_2_O, 84 mM Na_2_HPO_4_), fixed in 4% paraformaldehyde for 15 min at RT, and treated with 0.1% Triton X-100 in PBS for 10 min at RT to permeabilize cell membrane. After several washes, cells were blocked with 10% FBS in PBS for 45 min at RT to avoid non-specific binding, and then incubated with mAb against β-III tubulin (ab52623, Abcam, Cambridge, UK) at 1:50 dilution and at 4°C overnight. Next, cells were washed in PBS and incubated with goat anti-rabbit IgG H&L (Alexa Fluor 488) secondary antibody (ab150081, Abcam, Cambridge, UK) diluted 1:500 at RT for 1 h. After washing in PBS, cells were incubated for 15 min at 4°C in PBS with 15 μg/mL Hoescht (B2261, Sigma-Aldrich, Missouri, USA). Cover slips were washed in PBS, rinsed in distilled water and mounted on a slide with mounting medium for immunofluorescence detection (VectaShield, VectaStain, Vector Laboratories, California, USA). The samples were observed by laser-scanning confocal microscopy (Leica TCS SP5, Wetzlar, Germany), using 351/364 and 488 nm excitation lasers to visualize Hoescht and anti-β-III tubulin, respectively.

#### Trk receptor inhibition assay

To confirm that rrβ-NGF was in fact responsible for PC12 cell differentiation into neuron-like cells, a specific tyrosine kinase inhibitor (K-252a) was used to selectively block the effect of β-NGF in these cells. PC12 cells were incubated as described above and K-252a (Sigma Aldrich, Missouri, USA) was added 24 h post-seeding at 100 nM and the cells incubated for 2 h at 37°C. The following experimental groups were established: A) non-treated cells, B) cells treated with 25 ng/mL rrβ-NGF and, C) cells treated with 25 ng/mL rrβ-NGF and K-252a, as mentioned before. Differentiated cell percentages were determined after 48 h.

### Amino acid sequence of rabbit β-NGF

The full protein sequence of the rabbit prostate NGF (KX528686) generated was aligned with amino acid sequences of a few induced-ovulating species (*Lama lama*, *Camelus dromedarius*, *Camelus bactrianus*, *Vicugna pacos*) and spontaneous-ovulating species (*Rattus norvegicus*, *Mus musculus*, *Bos taurus*, *Homo sapiens*) using Clustal Omega Software. The glycosylation sites, disulfide bond sites, signal peptide, pro-peptide, beta chain and receptor binding sites indicated in the output results were compared among species.

### *In vitro* assessment of sperm viability and motility in the presence of rrβ-NGF

#### Animals, facilities and semen extraction

Six New Zealand White x California male adult rabbits, kept at the experimental farm of the Agrarian Production Department, Polytechnic University of Madrid (Spain), were used in this experiment. Animals were maintained in the same conditions as described above. On the farm, semen was collected weekly into an artificial vagina using a sexually receptive female.

#### Addition of rrβ-NGF to semen samples

After removing the gel fraction, the mass motility of each ejaculate was assessed and samples showing the highest values were pooled to a final mean concentration of 383.4 ± 71.4 x 10^6^ sperm/mL and subjected to different doses of rrβ-NGF depending on the experimental group. Doses were chosen according to β-NGF concentrations found in rabbit SP [7, 8, 10, 14]. The experimental groups established were: 0, 20 and 100 ng/mL, and 1 μg/mL (0, 1.5, 7.4 and 74.4 nM) of rrβ-NGF. In the 0 ng/mL group (negative control group), PBS was added instead of rrβ-NGF.

#### Sperm viability and motility analysis

Sperm viability and motility were assessed after 0, 1 and 2 h of rrβ-NGF challenge, time 0 being the moment of rrβ-NGF addition. At each time point, subsamples of semen were collected to assess sperm viability by nigrosin staining. Sperm motility was evaluated through Computer Assisted Semen Analysis (CASA), using the Motility module of the Sperm Class Analyzer (SCA) version 5.2 (Microptic S.L., Barcelona, Spain). A minimum of 200 sperm cells per experimental group was examined at each time point. The sperm motion parameters recorded were: percentage of static (STAT, %) and non-progressive sperm (NPMOT, %), velocity [curve-linear velocity (VCL, μm/s), straight-line velocity (VSL, μm/s), average path velocity (VAP, μm/s)] and percentages of linearity (LIN, %), straightness (STR, %) and wobble (WOB, %). This experiment was repeated 3 times.

### Ovulation induction in rabbit females inseminated with seminal doses containing rrβ-NGF

The semen of fertile adult rabbit bucks (n = 6) was collected using an artificial vagina, as described previously. White ejaculates free of gel were pooled together and diluted 1:5 (v:v) in a commercial rabbit extender (Inserbo S.L., Lérida, Spain) at a final concentration of 38.3 ± 7.1 x 10^6^ sperm/mL, assessed in a Neubauer chamber. Next, 28 adult female rabbits were randomly assigned to one of three groups to be artificially inseminated using a standard 22 cm-long curved plastic catheter: NGF (n = 10), inseminated with 1 μg/mL of rrβ-NGF in 0.5 mL of diluted semen; positive control (n = 10), injected intramuscularly with 20 μg/mL of gonadoreline (Inducel-GnRH, Lab. Ovejero, León, Spain) and inseminated with 0.5 mL of diluted semen; and negative control (n = 8), inseminated with an empty catheter. The dose of rrβ-NGF for this experiment was established according to the sperm motility results obtained (1 μg/mL) and was always added immediately before the AI time. Blood samples were collected in all females from the central ear artery at the moment of AI (time 0), and at 30, 60 and 120 min post AI to examine LH concentration, and at 0 and 7 days post AI to determine progesterone concentration, according to previous works [8]. Females were euthanized on Day 7 after AI and the following ovarian parameters were analyzed: ovulation rate [OR = (number of ovulating does/number of inseminated does) x100], number of corpora lutea (CL), number of hemorrhagic follicles (HF, follicles filled with blood and with no stigma indicating ovulation failure) and number of follicles larger than 1 mm (Fol>1mm). The presence of embryonic vesicles in the uterine horns was assessed to confirm pregnancy.

In addition, 8 females were inseminated with 1 μg/mL of rrβ-NGF in 0.5 mL of diluted semen as indicated above. Blood was drawn at the moment of AI (time 0), and at 1, 2, 3 and 4 h post AI to examine LH concentration, and at 0, 7 and 14 days post AI to determine progesterone concentration. In order to avoid the slaughter of an excessive number of animals, and according to the Ethics Committee, these 8 females were palpated to diagnosed pregnancy at 10 days post-AI and left them until delivery. The number of born alive and stillborn was recorded.

After blood collection, samples were centrifuged for 15 min at 700 x g and 4°C and the plasma stored at -20°C until analysis.

#### Hormone assays

Plasma LH concentration was determined by a homologous ELISA method validated at our laboratory [17]. Standards (from 400 to 0.781 ng/mL), zero standard (buffer), positive controls and plasma samples were run in duplicate. Inter-assay precision calculated in nine replicate measurements of coefficients of variation for pools of high and low concentrations was 3.1 and 6.8%, respectively.

For plasma P4 measurements, samples were extracted with petroleum ether at a 5:1 (v/v) ether:sample ratio (extraction efficiency was 85%) and analyzed using a competition ELISA kit (Progesterone ELISA, Demeditec Diagnostics GmbH, Kiel, Germany). Sensitivity was 0.045 ng/mL and intra- and inter-assay coefficients of variation were 5.5 and 6.9%, respectively.

Absorbance was measured in an Epoch microplate spectrophotometer (Bio-Tek, Vermont, USA) at 450 and 630 nm. Hormone concentration was calculated by extrapolation of a logistic five-parameter sigmoidal standard curve constructed using Master Plex software (USA).

### Statistical analysis

Data were analyzed using the SAS package version 9.0 (Statistical Analysis System Institute Inc, Cary, NC, USA). For the study of cell viability and neurite length of PC12 cells, a one-way ANOVA (GLM procedure in SAS) was used with β-NGF concentration (0, 5, 10, 25, 50 and 100 ng/mL) as the fixed effect. To examine differentiation and neurite outgrowth in PC12 cells over time, we performed a two-way ANOVA with repeated measures (MIXED procedure in SAS), with β-NGF concentrations (0, 5, 10, 25, 50 and 100 ng/mL) and examination days (2, 4, 6 and 8) as fixed effects, including also interaction between these two fixed effects in the statistical model. The effect of rrβ-NGF concentration on sperm viability and motility was assessed through two-way ANOVA with β-NGF concentration (0, 2, 20 and 100 ng/mL) and time (0, 1 and 2 h) as fixed effects, and again interaction between them was included in the statistical model. Finally, for the *in vivo* assays, two-way ANOVA with repeated measures was used to evaluate LH and P4 concentrations in all females, considering the experimental groups and the time of the analyses as fixed effects and including the interaction between these effects in the model. Ovarian parameters (number of CL, number of HF and number of Fol>1mm) were analyzed in ovulating and non-ovulating females. In an ANOVA, we then compared the number of HF and Fol>1 mm. Ovulation rates were compared in a χ2 test. All variables are provided as means ± SEM, and means were compared using Fisher test. Significance was set at p < 0.05.

## Results

### Recombinant rabbit β-NGF production and functional testing in PC12 cells

The complete nucleotide sequence of *NGF* from rabbit prostate was sequenced from its cDNA by the RACE procedure and submitted to the GenBank database under reference number KX528686.

Recombinant rβ-NGF was found expressed in the culture medium of CHO cells transfected with the plasmid pD2539-CEF-rβ-NGF. A single band of approximately 13–15 kDa was observed on Western blots. Further, the corresponding protein band extracted from the SDS-PAGE gel once examined by fingerprint analysis combined with mass spectrometry (MALDI-TOF) showed a high score for the rabbit *β-NGF* gene sequence (KX528686) inserted into the plasmid.

**Fig 1:**
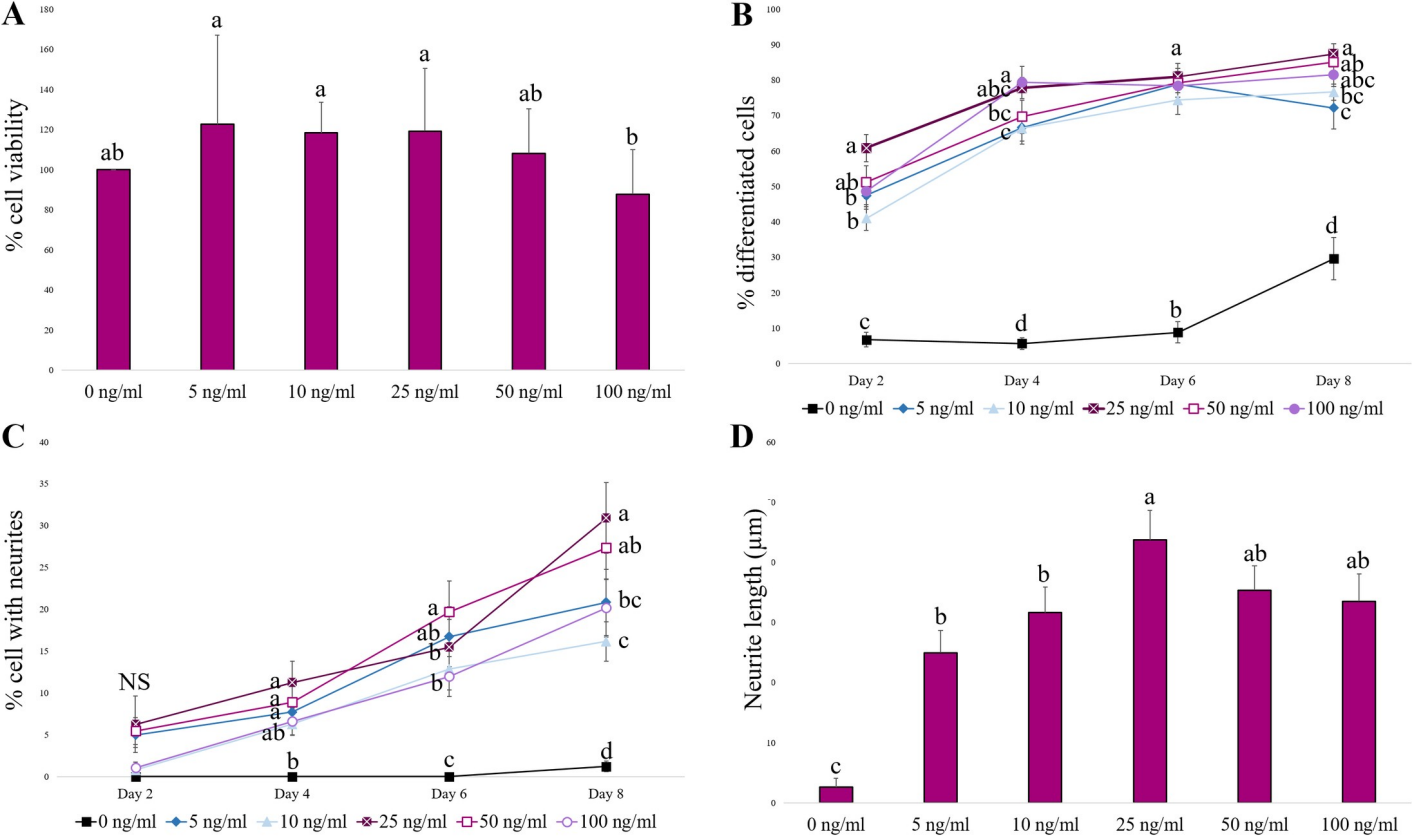
Dose-response study of different concentrations of rrβ-NGF (0, 5, 10, 25, 50 and 100 ng/mL) in PC12 cells. (A) Percentage viability of PC12 cells after 48 h of culture in the presence of different rrβ-NGF concentrations. Different letters indicate significant differences between doses tested (p<0.05). (B) Percentage of differentiated PC12 cells over time (until Day 8) in culture with different rrβ-NGF concentrations. Different letters indicate significant differences detected in the same day between doses tested (p<0.05). (C) Percentage of PC12 cells bearing neurites over time (until Day 8) in culture with different rrβ-NGF concentrations. Different letters indicate significant differences detected in the same day between doses tested (p<0.05). (D) Neurite lengths (μm) of PC12 cells on Day 8 of culture with different rrβ-NGF concentrations. Different letters indicate significant differences between doses tested (p<0.05). NS: non-significant differences. All data are represented as means ± SEM.

The cell viability of PC12 cells was similar for all doses of rrβ-NGF tested, except the highest dose (100 ng/mL). At this latter dose, a significantly lower percentage of viability was observed in comparison with rrβ-NGF doses of 5, 10 and 25 ng/mL (p<0.05), but similar to 50 ng/mL and 0 ng/mL doses which showed intermediate values (Fig 1A).

The percentage of differentiated PC12 cells was significantly higher in all the different rrβ-NGF concentration groups than negative control group at all time points examined (Fig 1B, p<0.05). On Day 2, the 25 ng/mL dose of rrβ-NGF gave rise to the highest percentage of differentiated cells, followed by 50 ng/mL. On Day 4, cells treated with 25, 50 or 100 ng/mL showed the greatest differentiation, whereas the 10 ng/mL concentration group displayed the lowest, but this was still higher than the percentage observed in the negative control. On Day 6, all groups showed the same differentiation percentage. At the end of the experiment (Day 8), the highest rate of differentiated cells was found again in the 25, 50 and 100 ng/mL rrβ-NGF groups.

The percentage of cells with at least one neurite was not significantly different on Day 2 (Fig 1C). However, by Day 4, in the 5, 25 and 50 ng/mL rrβ-NGF groups, a higher percentage of cells with neurites was recorded than in the 0 ng/mL group, whereas the 10 and 100 ng/mL groups showed intermediate values. On Day 6 the percentage of cells with neurites was higher in the 50 ng/mL group than all others, and the 5 ng/mL group featured intermediate values followed by the remaining rrβ-NGF treatment groups. On Day 8, cells treated with 25 ng/mL rrβ-NGF had the highest proportion of neurites, followed by those treated with 50 ng/mL of the neuropeptide.

Further, all the rrβ-NGF treatment groups showed longer neurites on Day 8 than the negative control group (0 ng/mL dose). The average length of neurites was significantly greater in the 25 ng/mL group than in the lower concentration groups (5 and 10 ng/mL), while cells treated with 50 and 100 ng/mL rrβ-NGF showed intermediated average lengths (Fig 1D).

**Fig 2:**
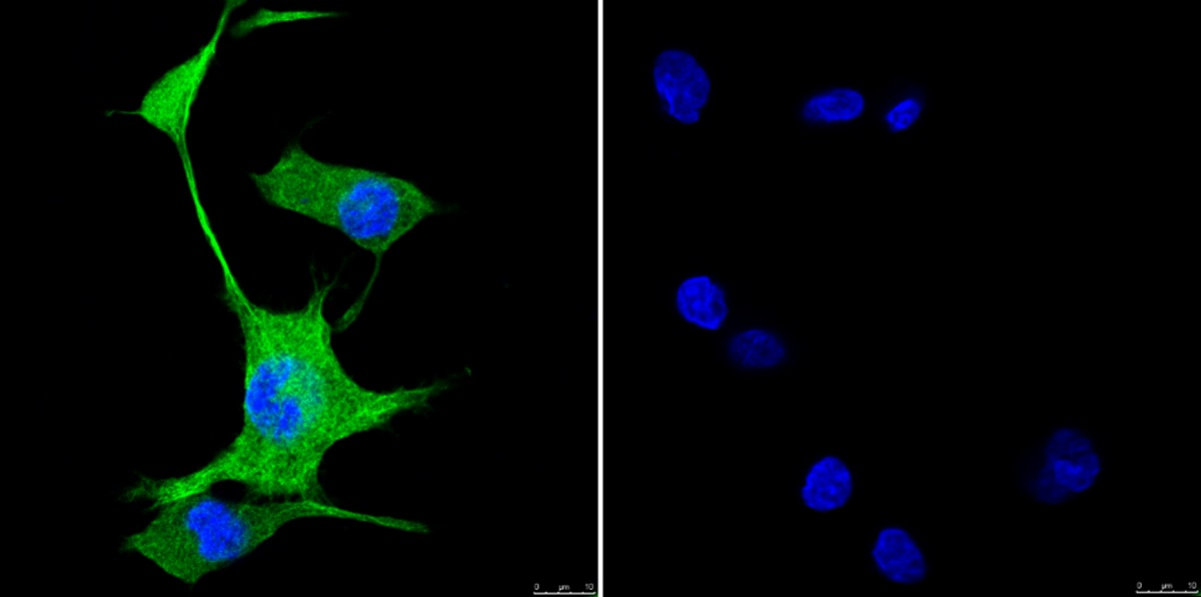
Immunodetection of β-III tubulin in PC12 cells treated with 25 ng/mL of rrβ-NGF on Day 8 of *in vitro* culture. Green signal represents binding to β-III tubulin (soma and neurites) and blue signal shows the nucleus stained with Hoescht 33342. Right panel is the negative control (lacking the primary antibody).

PC12 cells treated with 25 ng/mL of rrβ-NGF were positive for β-III tubulin immunofluorescence after 8 days of treatment (Fig 2) when high fluorescence was detected throughout the cytoplasm of the soma and in neurites. Cells cultured without rrβ-NGF showed no β-III tubulin expression.

**Fig 3:**
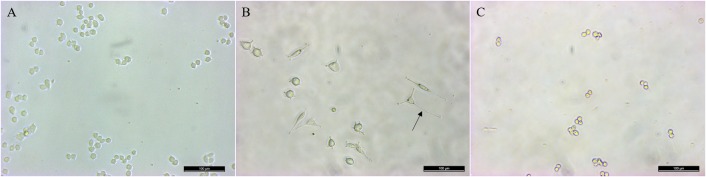
*In vitro* inhibition of the TrkA receptor in PC12 cells cultured with k-252a. (A) Non-treated cells (negative control). (B) Cells treated with 25 ng/mL rrβ-NGF (positive control). (C) Cells treated with K-252a+25 ng/mL rrβ-NGF. Arrow: neurite. Scale bar: 100 μm.

Finally, in the inhibition assay, PC12 cells showed no differentiation or neurite growth after 48 h of culture in conditions of co-treatment with K-252a plus rrβ-NGF (Fig 3). The percentage of cell differentiation in the positive control group was significantly higher than in the negative control group (72.21±1.00 *vs*. 20.51±2.81%, respectively, p<0.05).

### β-NGF protein sequence comparison among species

**Fig 4:**
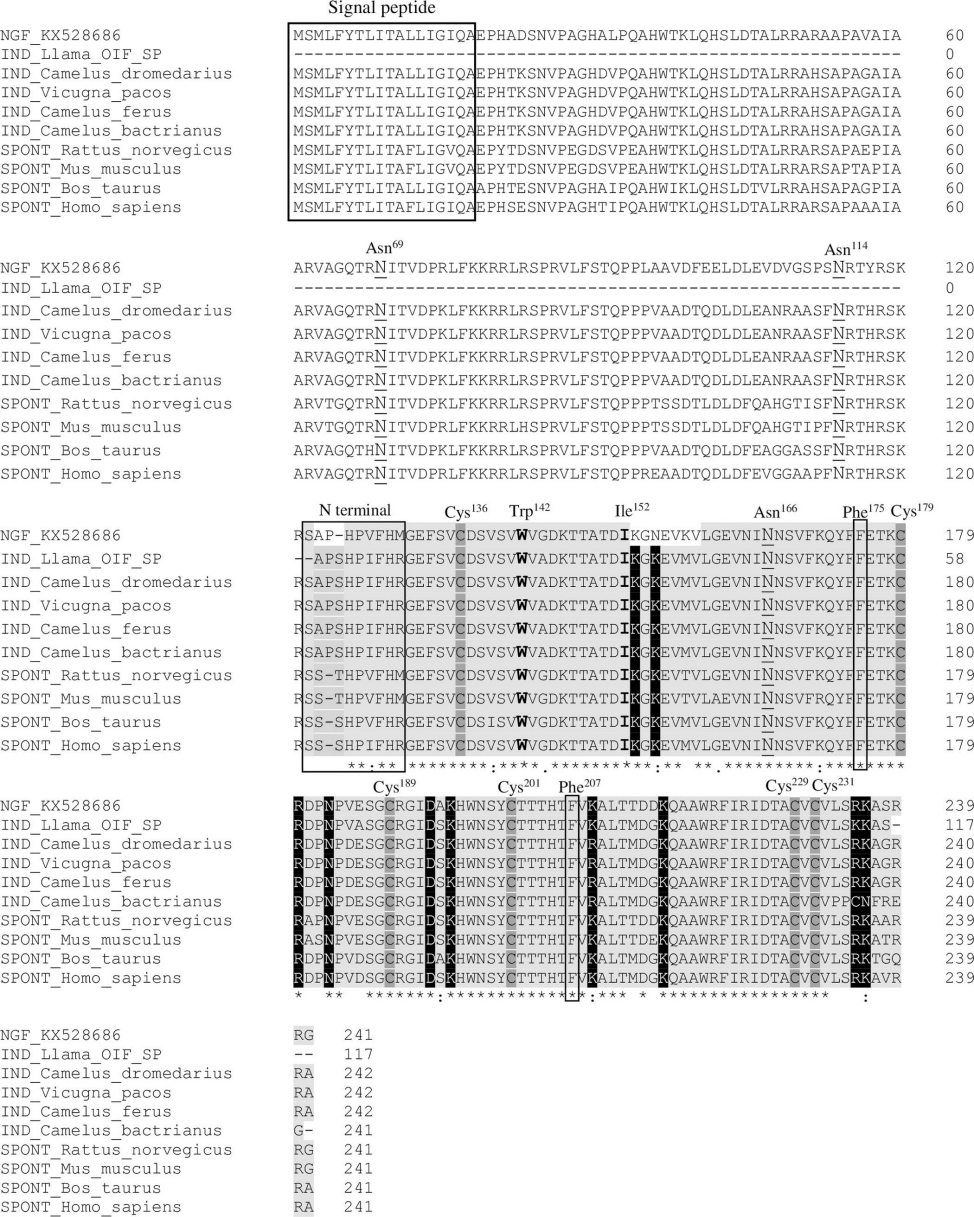
Amino acid sequence alignment of β-NGF in induced- and spontaneous ovulating species. IND (induced-ovulating species). NGF_KX528686: *Oryctolagus cuniculus*, KX528686; Llama_OIF_SP: ovulation inducing factor from SP of *Lama lama*, 4EFV_B; *Camelus dromedarius*, XP_010979007.1; *Vicugna pacos*, XP_015102944.1; *Camelus bactrianus*, XP_010967135.1. SPONT (spontaneous-ovulating species). *Rattus norvegicus*, NP_001263984.1; *Mus musculus*, NP_001106168.1; *Bos Taurus*, NP_001092832.1; *Homo sapiens*, NP_002497.2. The signal peptide is indicated in a box. Underlying residues (N^69^, N^114^ and N^166^) show glycosylation sites of Pro-NGF. The beta chain of β-NGF appears in light grey. Cysteines involved in disulfide bonds are indicated in dark gray (Cys^136^, Cys^179^, Cys^189^, Cys^201^, Cys^229^, Cys^231^). Amino acids that participate in both TrkA and p75 binding appear in bold (Trp^142^ and Ile^152^). TrkA binding sites are indicated in black boxes (N-terminal, Phe^175^, Phe^207^). P75 binding sites appear in white and highlighted in black. The differences observed in the rabbit beta chain are indicated in white.

Amino acid alignments of rrβ-NGF revealed conservation across the species examined of the signal peptide, 3 glycosylation sites (Asn^69^-Ile^70^-Thr^71^, Asn^114^-Arg^115^-Thr^116^, Asn^166^-Asn^167^-Ser^168^), all cysteines comprising 3 disulfide bonds (Cys^136^ –Cys^201^, Cys^179^ –Cys^229^, Cys^189^ –Cys^231^) and Trp^142^ and Ile^152^, which are important for TrkA and p75 binding (Fig 4). In induced-ovulating species evaluated (*Lama lama*, *Camelus dromedarius*, *Camelus bactrianus*, *Vicugna pacos*), the N-terminal region of the beta chain of NGF, where β-NGF binds to its high-affinity receptor, contained the tandem Ala-Pro, whereas in spontaneous ovulators there was a corresponding Ser residue. Further, specifically in the rabbit, the Ser after Ala-Pro identified in the remaining induced-ovulating species was missing. The majority of amino acids related to binding to p75 were conserved among species, except a sequence that was slightly different: KGNEVKVL in rabbit *versus* KGKEVMVL in the rest of the species examined.

### Effect of rrβ-NGF on rabbit sperm cells

**Fig 5:**
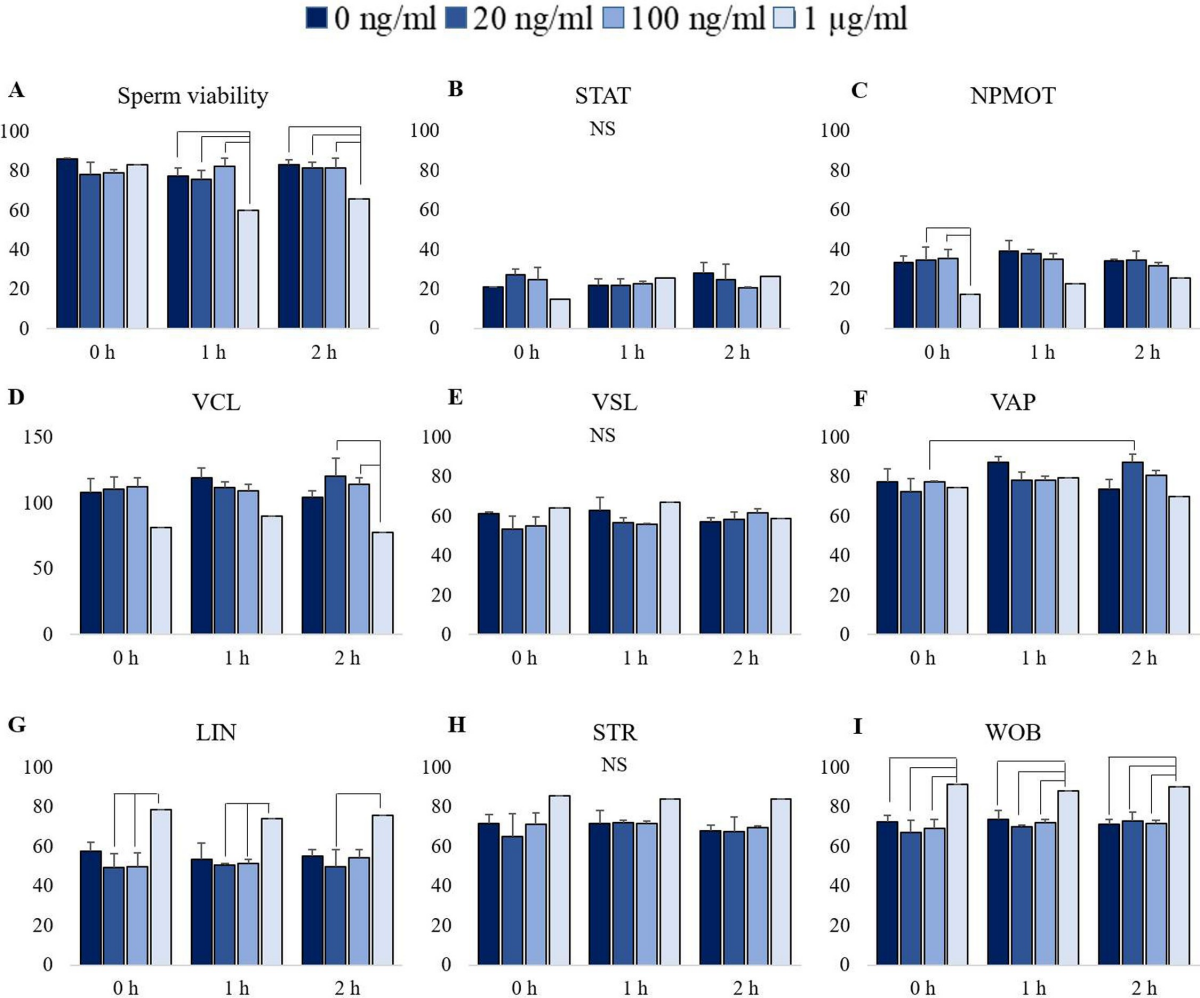
**Sperm viability (A) and motility parameters assessed by CASA (B-I) at 0, 1 and 2 h post addition of different doses of rrβ-NGF (0, 20, 100 ng/mL, 1** μ**g/mL) to fresh rabbit semen samples**. Concentrations of rrβ-NGF (0, 20, 100 ng/mL, 1 μg/mL) are represented in graded blue. Statistical differences among different concentrations at each time point are indicated by lines (p<0.05) except for VAP, whose line indicates differences between times. NS: non-significant differences. Data are represented as means ± SEM.

When rrβ-NGF was added to the semen samples (0 h), sperm viability was maintained at all doses tested (Fig 5A). After 1 and 2 h, semen treated with 1 μg/mL of rrβ-NGF showed a significant reduction in viability (p<0.05) compared with the other groups. Conversely, the percentage of static sperm (Fig 5B) was not different among groups at all time-points tested. The percentage of non-progressive sperm (NPMOT, Fig 5C) was similar in all the rrβ-NGF groups compared to the control group. Nevertheless, NPMOT was significantly lower in the 1 μg/mL-rrβ-NGF group than in the other treatment groups at the time of addition. There were no differences in velocity parameters (VCL, Fig 5D; VSL, Fig 5E; VAP, Fig 5F) for all concentrations and time points, except for VCL. Compared to the other treatment groups, this parameter showed lower percentages for the highest dose of the neurotrophin at 2 h. In addition, VAP was slightly elevated at 2 h in the 20 ng/mL concentration group. Finally, CASA parameters related to motion progression (LIN, Fig 5G; WOB, Fig 5I) were higher for the highest concentration of rrβ-NGF (1μg/mL) at all time points while sperm straightness did not vary (Fig 5H).

### Ovulation induction and ovarian response in rabbits when rrβ-NGF is added to the insemination dose

As depicted in Table 3, all females of the positive control group (10/10) and only 12.5% (1/8) of the negative control group ovulated, while the OR recorded in NGF-females 7 days post-AI was 60% (6/10), which is an intermediate rate between those of the other groups (p<0.05). In all NGF-treated does that ovulated, mean CL numbers were similar to the numbers recorded in the GnRH-treated females. No significant differences emerged between positive and negative controls and the NGF groups in numbers of HF or Fol>1 mm in any of the females (non-ovulating and ovulating).

**Table 3:** Ovarian parameters recorded in rabbit does treated with GnRH i.m. and inseminated (positive control), inseminated with 1 μg/mL rrβ-NGF (NGF group), or inseminated with an empty catheter (negative control).

	Positive control	NGF	Negative control
**Ovulation rate (%)**	100 (10/10) ^a^	60 (6/10) ^b^	12.5 (1/8) ^c^
**CL / ovulating doe**	13.3±0.47	12.67±1.31	19
**HF / ovulating doe**	1.4±0.43	0.5±0.22	1
**HF / non-ovulating doe**	-	0.75±0.25	0.86±0.7
**Fol>1mm / ovulating doe**	16.7±2.89	23±1.86	9
**Fol>1mm / non-ovulating doe**	-	21.75±4.91	14.71±0.84

CL: corpora lutea

HF: hemorrhagic follicles

Fol>1mm: follicles greater than 1 mm

Different superscripts in the same row indicate a significant difference (p<0.05)

Data represented as mean ± SEM

**Fig 6:**
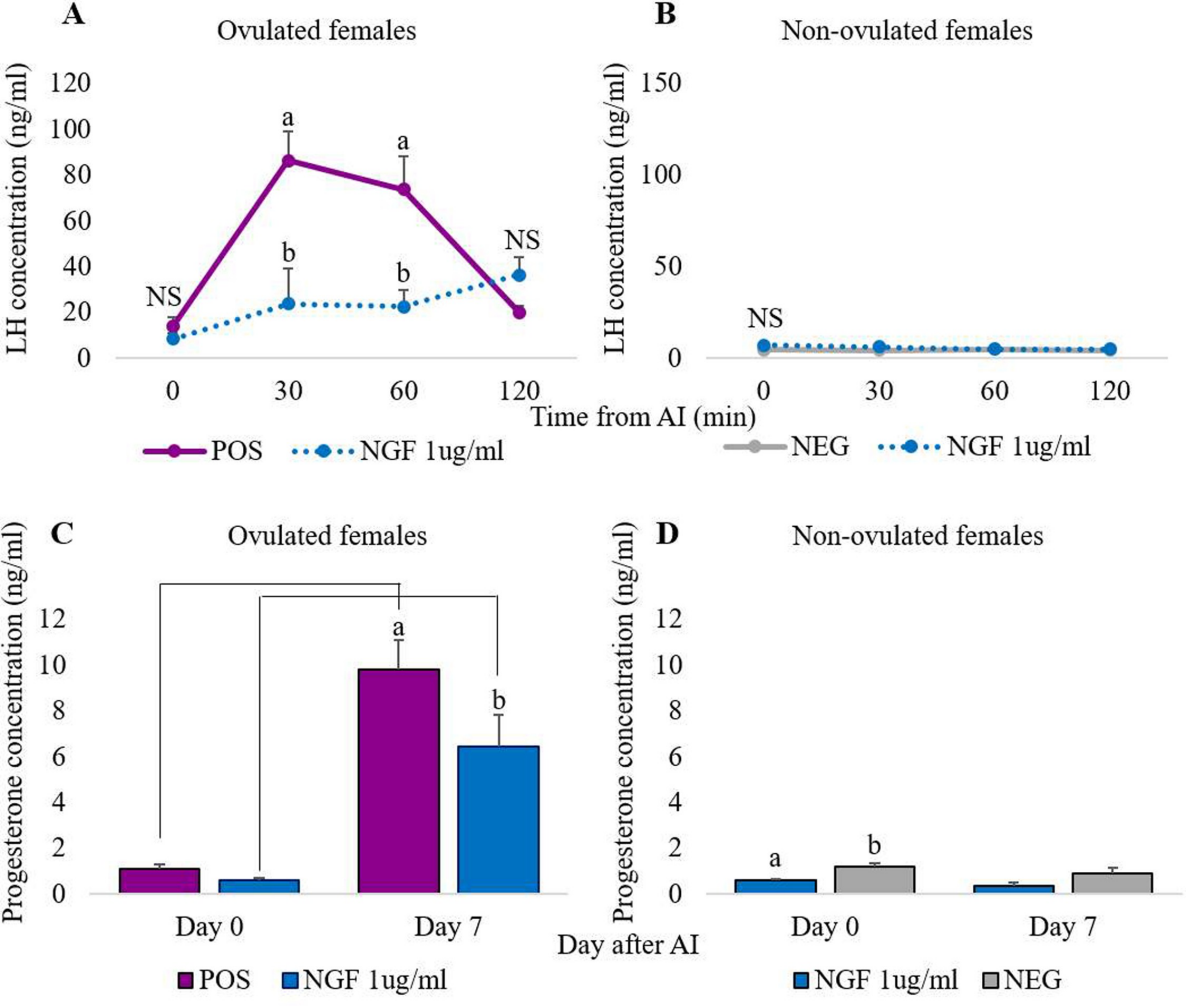
LH and progesterone concentration in ovulating and non-ovulating females treated with GnRH i.m. and inseminated (positive control), inseminated with 1 μg/mL rrβ-NGF (NGF group), or inseminated with an empty catheter (negative control). (A) LH concentration in ovulating females (positive control and NGF groups). (B) LH concentration in non-ovulating females (negative control and NGF groups). NS: non-significant difference. Significant differences at the same time-point are indicated by different letters (p<0.05). Data are represented as mean ± SEM. (C) Progesterone concentration in ovulating females (positive control and NGF groups). (D) Progesterone concentration in non-ovulating females (NGF and negative control groups). Significant differences between groups are indicated by letters and between days by lines (p<0.05).

**Fig 7:**
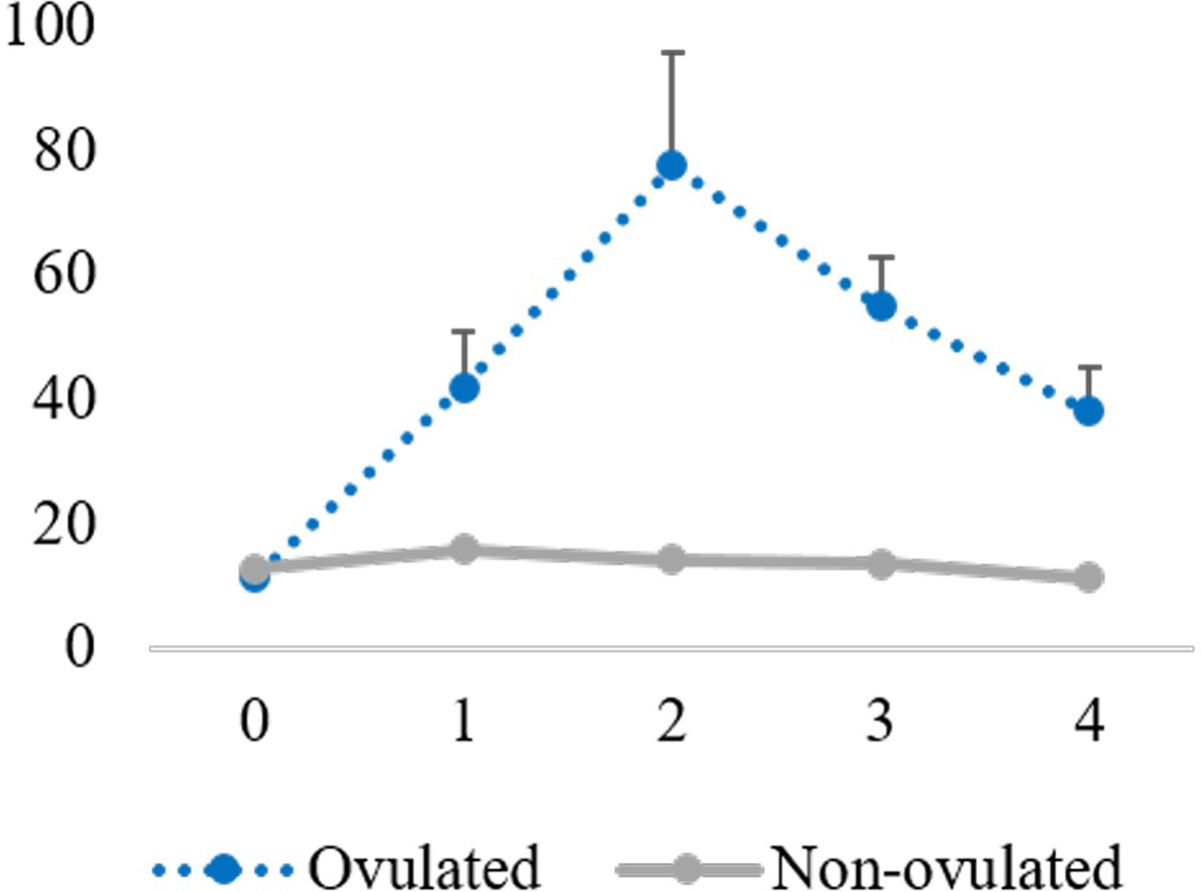
LH concentration assessed during 4 h after AI, in ovulating and non-ovulating females treated with 1 μg/mL rrβ-NGF.

In ovulating females, plasma LH concentration increased in the positive control group peaking 30–60 min after AI while in the NGF group, LH levels remained lower (p<0.05) than those recorded in the positive control females at these time points. At 120 min, LH sharply fell in the GnRH-treated females to similar levels to those found in the NGF-treated does (Fig 6). Non-ovulating females showed baseline levels of LH that remained stable from 0 to 120 min. When analyzing LH concentration for 4 h on ovulating females, LH progressively increased, reaching the maximum 2 hours after AI. LH levels decreased gradually 3 and 4 h after AI (Fig 7). Non-ovulating females showed no increase in LH concentration.

Progesterone levels significantly increased (p<0.05) from Day 0 to Day 7 in all ovulating females in the GnRH and NGF groups (Fig 6C), and were significantly higher on Day 7 (p<0.05) in the GnRH-treated females than in the NGF-treated does. In non-ovulating does (Fig 6D), P4 concentration did not increase on Day 7, and was higher in negative control females than in NGF-treated rabbits on Day 0; on Day 7, levels were similar. Embryonic vesicles in the uterine horns were observed in all ovulating females at day 7, except in the only female that ovulated in the negative control group. Fifty percent (4/8) of the second group of NGF-treated does became pregnant and in them, the progesterone also increased from Day 0 to Day 7 of pregnancy (0.4±0.01 to 14.0±1.1 ng/ml, respectively). The number of born alive and stillborn was 11.5±0.5 and 0.3±0.3, respectively.

## Discussion

In this study, we sequenced *NGF-*mRNA isolated from rabbit prostate, and produced and purified a novel recombinant rabbit β-NGF protein. We also confirmed the biological effects of this protein as it was able to induce the differentiation of PC12 cells into neuron-like cells. When the amino acid sequence of rabbit NGF was compared with those of other induced-ovulating or with spontaneous-ovulating species, species-specific differences emerged mainly in their receptor binding sites. The protein was also found to have a dose- and time-response effect on rabbit sperm viability and motility parameters. Finally, when added to the insemination dose, ovulation occurred in rabbits after a delayed LH peak and this was followed by a physiological P4 rise, conception and delivery.

One of the challenges of recombinant protein production is to ensure the same post-translational modifications as those of the native proteins. These modifications are needed for the protein's biological activity and can be achieved by intracellular engineering of mammalian cells such as CHO cells [20]. In our study, transfection of these cells with a plasmid containing rabbit β-NGF led to the successful production of the native protein showing an appropriate molecular weight, amino acid sequence, and proper biological activity through the TrkA receptor. In the presence of rrβ-NGF, PC12 viability was maintained regardless of the concentration tested. Cells treated with 25 or 50 ng/mL of the protein featured the highest percentages of cell differentiation and neurite growth per cell throughout the study, and also showed more intense neurite outgrowth than described by others in the presence of 50 ng/mL of the NGF [45]. In contrast, Gunning et al. [46] noted that higher concentrations of mouse β-NGF than those used in the present study (150 ng/mL) could progressively increase the percentage of cells with neurites. Probably, the origin of β-NGF can affect PC12 cell differentiation due to different affinities to TrkA receptors [47].

To examine the physiological particularities of rabbit β-NGF, we compared its protein sequence with that of β-NGF obtained from other species. Several differences were identified in binding sites to its receptors. Hence, two consecutive Ala-Pro residues were found in the N-terminal region of rabbit β-NGF, as well as in all the induced-ovulating species examined. This region is important for binding to TrkA and our finding could indicate a different strength of binding to TrkA, since Pro has a particular structure that may facilitate a greater angle of torsion. Conversely, after this tandem of amino acids, a Ser residue was found in all the species studied, except the rabbit. This missing residue may be relevant to create a more stable configuration through the torsion facilitated by the previous Pro. Further, two amino acid residues, which participate in recognition of the low-affinity receptor p75 [48], had mutations in the rabbit sequence. Thus, Asn^155^ substituted the conserved Lys, and Lys^158^ also replaced a conserved Met. The Lys residue has a positive charge and repels the protonated histidine within the binding site of p75. Its replacement with an Asn residue, which has a neutral charge, could promote approximation of β-NGF to its low-affinity receptor. We recently reported the expression and localization of TrkA [10] and p75 [9] in the male rabbit genital tract, supporting its likely role in rabbit reproduction. Despite such meaningful differences in the amino acid sequences of binding domains to receptors, some biological functions of rabbit β-NGF were conserved in other species. Accordingly, in llama females, rabbit SP produced ovulation as efficiently as llama SP [3] such that interaction with the receptor does not seem to be modified in other species. However, llama SP is not able to elicit ovulation in rabbits [3], suggesting that these specific residues found in the rabbit amino acid sequence are needed for some of the particular physiological characteristics of the rabbit ovulation process. Notwithstanding, the different sexual stimulation required to trigger ovulation and numerous SP components in both these species have to be considered. Further work is needed to clarify the mechanisms of rabbit β-NGF function in the female reproductive tract.

Since β-NGF is present in the SP of several species and its high- and low-affinity receptors have been found in sperm cells of bovine [33], human [49] and llama [50], the addition of a certain dose of β-NGF in semen could presumably exert a physiological effect on sperm, yet the presence of this neurotrophin in rabbit sperm cells is not known. To determine the best concentration of β-NGF not deleterious for sperm, and with optimal effect on the viability and motility, we tested different concentrations of the recombinant protein on raw rabbit semen. In contrast with other studies, where sperm viability was enhanced 2 h after the addition of NGF in a range from 20 to 120 ng/ml (bovine frozen sperm, [31]) or 30 min after adding 0.5 and 1 ng/ml of NGF (human fresh semen [30]), this parameter was maintained on almost every concentration tested. However, in the current study the addition of 1 μg/mL rrβ-NGF diminished sperm viability 1 or 2 h after challenge. Our results related to velocity parameters (VCL, VSL, VAP) also disagreed with the literature. While these parameters were enhanced in fresh human semen at 10 μM of NGF [30, 32] and in caudal epididymis sperm of golden hamster at 500 and 1000 ng/ml [34], our results showed only differences at 2 h in 1 μg/ml-rrβ-NGF concentration. Contradictory, the parameters related with the sperm progressivity improved immediately with the addition (NPMOT, LIN, WOB) or 1 and 2 h after (LIN, WOB) when semen was treated with 1 μg/ml of rrβ-NGF. These findings partially support the literature reviewed [30, 32, 34]. These differences found with the previous works could be due to the sample nature (fresh *vs*. frozen or raw *vs*. diluted semen), the NGF concentration and time of treatment, or the specific-species mechanism of NGF in sperm cells. It is noteworthy that the present study was done in the rabbit, a reflex ovulator, and the literature reported, to the best of our knowledge, these effects only in spontaneous ovulators (golden hamster [34], bovine [33], human [30–32]). Our results suggest that NGF has effect on rabbit sperm cells when it is in a high concentration (1 μg/ml), much higher than the physiological concentration found in rabbit SP of around 2 ng/mL [10, 14]. The improvement of progressivity parameters with the addition of the neurotrophin at time 0 mentioned in this work are interesting since they are important indicators of the sperm’s ability to fertilize an egg [34]. Further studies of localization of NGF and its receptors in rabbit sperm, or blocking the specific receptors of the neurotrophin if they are present, are necessary to elucidate the physiology of this mechanism.

Based on our sperm viability and motility results, and consistent with the β-NGF concentration found in rabbit SP [7, 8, 10, 14], we selected a 1 μg/mL concentration for its addition to the insemination dose. This dose led to ovulation, embryo implantation and gestation of the does inseminated with semen containing rrβ-NGF. As far as we know, this is the first report of ovulation in response to NGF in the insemination dose administered via the intravaginal route in rabbits. In other studies, no evidence [3,16] or low rate [8] of ovulation was detected when rabbit SP or mouse β-NGF, respectively, were administered intramuscularly to rabbits, suggesting that maybe the origin and purity of the protein is important for the mechanism of ovulation in rabbits. In our study, the ovulation rate of females treated with rrβ-NGF, nevertheless, was not 100% as in the GnRH group. The mechanism of NGF in ovulation induction remains unknown. We hypothesize this could be because the neurotrophin can act together with another component of the SP to induce ovulation, in agreement with that proposed by Rebollar et al. [17]. Another explanation for not reaching 100% of ovulation in this study after NGF administration may be the route and the dose used. Previous studies have reported the requirement of higher doses of analogues of GnRH via the intravaginal route to achieve an OR of 100% [17, 35–37]. Similarly in this study, it may be necessary to increase the dose or to change the route of administration to reach a higher OR. Further experiments of *in vivo* dose-response studies using this recombinant protein are required to clarify the mechanism of action of the neurotrohpin.

In the current work, the LH peak was not observed in the ovulating females in the first experiment performed, while it was detected in the GnRH group from 30 to 60 min after AI, according with our previous works [17, 37, 51, 52]. Therefore, an additional 8 females were used, extending the time of LH concentration analysis up to 4 h after AI. We performed this new group using the least possible number of animals. Thus, only the NGF group was repeated, since the LH concentration of GnRH group until 3, 7 h and even 5 days has been reported previously by our group [37, 51, 52]. The results showed a delayed in LH surge in does treated with 1 μg/ml rrβ-NGF, presenting maximum values 2 hours after AI. Previous studies in camelids have reported this delay in animals treated with SP or NGF obtained from SP [53, 54] or when GnRH analogues are administered by intravaginal route [37]. The explanation for this delay in the LH peak is unclear, although Adams et al [55] suggested it could be the consequence of an unknown intermediate step in the ovulation pathway required for GnRH/LH release because the regulation of GnRH secretion in neurons depends on numerous inputs [56]. One of these inputs can be provided by interneurons such as kisspeptins and noradrenergic neurons after NGF interaction, as postulated by Carrasco et al. [57].

Here, firstly we observed CL formation and increased plasma P4 levels in ovulating rrβ-NGF-females from Day 0 to 7, which shows that the LH peak and ovulatory response occurred in 60% of does. Once formed, the CL releases progesterone and is the primary source of this hormone in the rabbit female during gestation [58]. However, despite having the same number of CL, ovulating does treated with β-NGF had lower plasma concentrations of progesterone compared to the GnRH-treated females, as occurs in females treated intravaginally with analogues of GnRH [37]. The intravaginal administration of substances requires its absorption through the vaginal mucosa, thus presumably higher doses via intra vaginal would be necessary to achieve the same P4 concentration as when administering via i.m. However, it cannot be disregarded that this lower P4 levels in NGF-group could be the consequence of a reduced luteotrophic effect of the neurotrophin in rabbits, being lower compared to the reported effect of β-NGF in camelids [54, 59]. The possible reason of this finding could be the fact that the NGF provokes a last-longer LH surge in camelids compared to rabbits, which results in the formation of a greater CL and consequently a release of higher P4 concentrations [53]. Despite having lower P4 concentration than GnRH-treated females, all rrβ-NGF females were diagnosed as pregnant and they achieved a normal number of born alive, not being reported when analogues of GnRH were used intravaginally [38], which suggests that rrβ-NGF could promote sufficient CL luteinization to be functional. Accordingly, the concentration of P4 reported in this work was within standard levels reported by previous studies in pregnant does [60]. In addition, in this study, when testing a higher dose than found in SP, β-NGF did not affect fertility, early embryo development/implantation, gestation and delivery. Indeed, studies have shown that β-NGF enhances embryo development *in vitro* [61]. We observed only one ovulating doe (but not pregnant) in the negative control group. This is most likely attributable to the nervous stimulus of insemination catheter insertion at the time of AI, as previously reported [17].

In conclusion, the new recombinant rabbit β-NGF produced in CHO cells is a functional protein with biological effects. This was confirmed in cultured PC12 cells by its capacity to induce their differentiation, the appearance of β-III tubulin in the cells and the absence of neurite growth in the presence of a TrkA inhibitor. The protein also features unique amino acid residues at receptor binding sites, which could explain some of the particularities of the reproductive physiology of the rabbit. Exogenous rrβ-NGF supplementation of ejaculated rabbit sperm did not affect sperm viability at the moment of its addition and was found to have a dose- and time-dependent effect on rabbit sperm viability and motility parameters. When the neuropeptide was added to the seminal dose before AI, it was able to elicit ovulation followed by a P4 rise and gestation. Further studies with this new recombinant protein must be done to enhance our understanding with the aim to be intravaginally administered to rabbit does to induce ovulation at the time of the AI. This could improve breeding systems in this species avoiding the use of high concentrations of synthetic analogous by the intravaginal via. The delay in the LH peak observed in rabbit females treated with rrβ-NGF needs further investigation.
