# Correction: Recombinant rabbit beta nerve growth factor production and its biological effects on sperm and ovulation in rabbits

**DOI:** 10.1371/journal.pone.0223091

**Published:** 2019-09-23

**Authors:** Ana Sanchez-Rodriguez, Paloma Abad, María Arias-Alvarez, Pilar Millán, Pilar G. Rebollar, José M. Bautista, Pedro L. Lorenzo, Rosa M. García-García

Pilar Millán should be included in the author byline instead of the Acknowledgments. Pilar Millán should be listed as the fourth author, and their affiliation is 1: Department of Physiology, Faculty of Veterinary Sciences, Complutense University of Madrid, Madrid, Spain. The contributions of this author are as follows: Formal analysis, Methodology, Resources, Writing–Review & Editing.

The correct citation is as follows: Sanchez-Rodriguez A, Abad P, Arias-Alvarez M, Millán P, Rebollar PG, Bautista JM, et al. (2019) Recombinant rabbit beta nerve growth factor production and its biological effects on sperm and ovulation in rabbits. PLoS ONE 14(7): e0219780. https://doi.org/10.1371/journal.pone.0219780
